# Phosphoproteomics reveals network rewiring to a pro-adhesion state in annexin-1-deficient mammary epithelial cells

**DOI:** 10.1186/s13058-017-0924-4

**Published:** 2017-12-12

**Authors:** Asfa Alli-Shaik, Sheena Wee, Lina H. K. Lim, Jayantha Gunaratne

**Affiliations:** 1 Translational Biomedical Proteomics, Institute of Molecular and Cell Biology, Agency for Science, Technology and Research, 61 Biopolis Drive, Singapore, 138673 Singapore; 2 Department of Physiology, Immunology Programme, Centre for Life Sciences, Yong Loo Lin School of Medicine, National University of Singapore, 28 Medical Drive, Singapore, 117456 Singapore; 3 Department of Anatomy, Yong Loo Lin School of Medicine, National University of Singapore, 10 Medical Drive, Singapore, 117597 Singapore

**Keywords:** Annexin-1, Breast cancer, Proteomics, Phosphoproteomics, Network analysis, Adhesion, Mammary epithelial cells

## Abstract

**Background:**

Annexin-1 (ANXA1) plays pivotal roles in regulating various physiological processes including inflammation, proliferation and apoptosis, and deregulation of ANXA1 functions has been associated with tumorigenesis and metastasis events in several types of cancer. Though ANXA1 levels correlate with breast cancer disease status and outcome, its distinct functional involvement in breast cancer initiation and progression remains unclear. We hypothesized that ANXA1-responsive kinase signaling alteration and associated phosphorylation signaling underlie early events in breast cancer initiation events and hence profiled ANXA1-dependent phosphorylation changes in mammary gland epithelial cells.

**Methods:**

Quantitative phosphoproteomics analysis of mammary gland epithelial cells derived from ANXA1-heterozygous and ANXA1-deficient mice was carried out using stable isotope labeling with amino acids in cell culture (SILAC)-based mass spectrometry. Kinase and signaling changes underlying ANXA1 perturbations were derived by upstream kinase prediction and integrated network analysis of altered proteins and phosphoproteins.

**Results:**

We identified a total of 8110 unique phosphorylation sites, of which 582 phosphorylation sites on 372 proteins had ANXA1-responsive changes. A majority of these phosphorylation changes occurred on proteins associated with cytoskeletal reorganization spanning the focal adhesion, stress fibers, and also the microtubule network proposing new roles for ANXA1 in regulating microtubule dynamics. Comparative analysis of regulated global proteome and phosphoproteome highlighted key differences in translational and post-translational effects of ANXA1, and suggested closely coordinated rewiring of the cell adhesion network. Kinase prediction analysis suggested activity modulation of calmodulin-dependent protein kinase II (CAMK2), P21-activated kinase (PAK), extracellular signal-regulated kinase (ERK), and IκB kinase (IKK) upon loss of ANXA1. Integrative analysis revealed regulation of the WNT and Hippo signaling pathways in ANXA1-deficient mammary epithelial cells, wherein there is downregulation of transcriptional effects of TEA domain family (TEAD) suggestive of ANXA1-responsive transcriptional rewiring.

**Conclusions:**

The phosphoproteome landscape uncovered several novel perspectives for ANXA1 in mammary gland biology and highlighted its involvement in key signaling pathways modulating cell adhesion and migration that could contribute to breast cancer initiation.

**Electronic supplementary material:**

The online version of this article (doi:10.1186/s13058-017-0924-4) contains supplementary material, which is available to authorized users.

## Background

Annexin-1 (ANXA1), also known as lipocortin-1, belongs to a multigene superfamily of calcium-dependent phospholipid binding proteins that regulate inflammatory responses [1, 2]. ANXA1 is a prime effector of anti-inflammatory effects of glucocorticoids wherein it inhibits phospholipase A2 to suppress eicosanoid production such as prostaglandins, and also leukocyte functions [3]. Apart from controlling production of proinflammatory mediators, ANXA1 also limits neutrophil recruitment and promotes phagocytosis of apoptotic neutrophils [4, 5]. While the roles of ANXA1 in innate immune responses are well-established, growing evidence of its functions in adaptive responses that has emerged in recent years, however, has been inconsistent [6]. While some reports indicate that ANXA1 increases T cell activation and differentiation, others focus on ANXA1-mediated attenuation of T cell responses [7, 8].

Besides the inflammatory effects, ANXA1 also plays essential roles in cell proliferation, differentiation, apoptosis and cancer [2]. By modulating the functions of several receptors including epidermal growth factor, ANXA1 influences several downstream signaling cascades [9, 10]. With its contributions prominent in a wide array of cellular functions, ANXA1 either functions as a pro-cancerous or tumor-suppressive protein depending on the contextual tumor tissue/cell type. In several malignancies associated with liver, pancreas, lung, prostate and brain, high ANXA1 expression has been correlated with tumor progression, aggressiveness and even metastasis [11–15]. Despite ANXA1 being considered a prognostic marker, its status in tumor progression and survival in breast cancer remain contradictory [16–18]. In general, ANXA1-positive cases have been associated with clinically aggressive basal-like breast cancer. In these cells, ANXA1 activates an epithelial-mesenchymal switch (EMT) by enhancing transforming growth factor (TGF)β/Smad signaling thereby exacerbating migration and invasion [19]. With ANXA1 localizing at F-actin-rich membrane ruffles, a potential role in shaping the cytoskeletal network has also been proposed. Remodeling of the actin cytoskeletal network is instrumental in deciding cell fate towards migration and invasion [20]. Our previous studies emphasized that ANXA1 modulates cell adhesion and migratory properties during breast cancer initiation and tumorigenesis [21, 22]. However, the underlying signaling mechanisms for such modulations are not clear on a global scale. The spatial and temporal regulation of many proteins, including their activation status is more often determined by their phosphorylation status. Hence, here we extend our previous work by investigating the phosphorylation landscape in ANXA1^+/-^ and ANXA1^-/-^ murine mammary gland cells to understand the ANXA1-modulated signaling network upon breast cancer initiation. Our phosphorylation dataset complements our previous proteome profiling, and provides novel insights into several ANXA1-dependent processes including its role in rewiring adhesion-related machinery.

## Methods

### Cell lysate preparation

Stable isotope labeling with amino acids in cell culture (SILAC)-adapted cell lines previously established from mammary gland epithelial cells from ANXA1-heterozygous and ANXA1-deficient mice were used in this study [21]. The SILAC-adapted “heavy-labeled” and “light-labeled” cells were lysed with 4% SDS (w/v), 100 mM Tris/HCl pH 7.6 and 100 mM DTT at room temperature. Protein was quantified using the RCDC assay kit (Bio-Rad) prior to equal mixing of identically concentrated light and heavy SILAC-labeled ANXA1^+/-^ and ANXA1^-/-^ lysates.

### Reduction, alkylation and digestion

Filter-aided sample preparation (FASP) was performed as described with slight modifications [23]. In short, the sample was mixed with 5 mL UA buffer (8 M urea in 0.1 M Tris/HCl pH 8.5) and poly(ethylene) glycol (PEG) (with a final PEG concentration of 0.5%) in a 15-mL 30-kDa Amicon filter (Millipore) followed by centrifugation at 3850 × g for 45 minutes at room temperature. The retentate was rinsed with 4 mL of UA and followed by 2 mL of UA: 2 mL of 50 mM iodacetamide in UA was then added followed by incubation for 20 minutes in the dark and then by centrifugation to remove the iodoacetamide. Samples were washed one more time with 2 mL of UA, followed by three washes with 2 mL 40 mM ammonium bicarbonate. Sequencing-grade trypsin (Promega) in 40 mM ammonium bicarbonate was added. The trypsin to protein ratio was 1:100 (w/w). After overnight digestion at 37 °C the peptide solution was collected by centrifugation. The amount of peptide obtained was measured using Nanodrop (Thermo Scientific).

### Phosphopeptide enrichment

Phosphopeptides were enriched from the tryptic peptides as described with slight modifications [23]. Briefly, around 6 mg of FASP-digested tryptic peptides were diluted four times with 1 M glycolic acid/80% acetonitrile (v/v)/2% trifluoroacetic acid (TFA) (v/v) and added to 13 mg of Titansphere TiO_2_ 10 μm (GL Sciences, Inc., Japan). After incubating the mixture for 10 minutes at room temperature, the sample was centrifuged at 4000 g for 3 s. The supernatant was collected and the above process was repeated with another portion of the beads. The beads were washed four times with 1 M glycolic acid/80% acetonitrile (v/v)/2% TFA (v/v) solution and four times with 80% acetonitrile (v/v)/0.1% TFA (v/v) solution after transferring to a 200-μL pipet tip plugged with two layers of C8 empore disks (3 M Empore). Finally, the phosphopeptides were eluted using 500 uL 40% acetonitrile (v/v) containing 15% NH4OH (m/v) followed by vacuum-concentration to ∼ 40 μL.

### Anion exchange fractionation of phosphopeptides

Phosphopeptides were fractionated as described, with slight modifications [24]. Briefly, phosphopeptides were diluted five times in Britton & Robinson buffer composed of 20 mM CH_3_COOH, 20 mM H_3_PO_4_, 20 mM H_3_BO_3_ and NaOH, pH 11. The sample was then fractionated on a micropipette tip with six layers of 3 M Empore Anion Exchange disks (Varian, 1214-5012) stacked into it. Peptides were loaded at pH 11 and fractions were subsequently eluted with buffer solutions of pH 8, 6, 5, 4 and 3, respectively into their respective C18 stage tips.

### Mass spectrometry (MS)

Full-scan MS spectra corresponding to m/z 300–1400 were acquired using Orbitrap or Orbitrap XL (Thermo Fisher) for all samples with a resolution of *r* = 60,000 at m/z 400, an AGC target of 1e6, and a maximum injection time of 500 ms. For each scan, the top ten most intense peptide ions with intensity threshold >2000 and charge state ≥2 were isolated sequentially to a target value of 1e4, and the precursor ions were fragmented by collision-induced dissociation in a linear ion trap using normalized collision energy of 35%. Dynamic exclusion was applied for 30 s using a maximum exclusion list of 500.

### Identification and quantification of peptides and proteins

Data were processed using MaxQuant (Version 1.3.0.5) [25, 26] against uniprot 2013-01 mouse database containing 262 commonly observed contaminants. Raw mass spectrometry data corresponding to our previously published proteome profiling carried out under a similar experimental set up was also included for analysis. Database searches were performed allowing a maximum of two missed cleavages upon trypsin digestion, two labeled amino acids, and an initial mass tolerance of 6 ppm for precursor ions and 0.5 Da for fragment ions. Carbamidomethylation of cysteine was searched as a fixed modification, and N-acetylation, oxidized methionine and phosphorylated serine/threonine/tyrosine were searched as variable modifications. Peptide and protein quantification of SILAC pairs was performed using default settings at a maximum false discovery rate (FDR) of 0.01. Only those proteins supported by at least one unique peptide with a minimum length of six amino acids were considered to be identified.

### Differential expression analysis

For differential expression analysis only those phosphosites that can be localized with a localization probability >0.75 were considered. The SILAC ratios of the corresponding peptides were log-transformed, and each replicate was median-centered and scaled for median absolute deviation (MAD). Only those phosphopeptides greater than the robust *z* score threshold of 2 or less than -2 (approximately equating to at least 1.8-fold change in this dataset) reliably in three out of four experiments were considered to be regulated. Functional enrichment for the regulated phosphoproteins was obtained as Gene Ontology (GO) terms corresponding to biological process, cellular component, and molecular function using the Database for Annotation, Visualization and Integrated Discovery (DAVID) functional annotation tool [27]. Only those categories containing at least three genes were considered for analysis. The GO molecular function categories that were significantly enriched with a *p* value <0.01 in either of the increased or decreased phosphorylation groups were visualized as a heatmap.

### Kinase mapping and enrichment

The upstream kinases of the regulated phosphosites were obtained by mapping the site-specific information against a comprehensive knowledge base of experimentally derived kinase-substrate relationships (KSR) available from KSR-LIVE [28]. The integrated database combines KSRs from multiple repositories such as PhosphoSitePlus, PhosphoELM, PhosphoPOINT and the Human Protein Reference Database (HPRD). The derived upstream kinases along with the regulated phosphoproteins were analyzed using the search tool for the retrieval of interacting proteins (STRING) to explore their functional connectivity [29]. Next, the network was filtered to retain only the high-confidence interactions. This filtered network was finally combined with kinase mapping information to build the ANXA1-specific signaling network. The network was rendered in Cytoscape [30].

Upstream kinase prediction was also carried out using the NetworKIN (version 3.0) algorithm that uses kinase consensus motif along with contextual modeling for predicting KSRs [31]. The mouse phosphopeptides were mapped to their corresponding human homologs and the respective upstream kinases were derived for all regulated phosphosites. Then kinase activity estimation was performed based on average abundance of the regulated phosphopeptide in the predicted kinase substrate set [32]. An activity score was computed for each kinase and the statistical significance of the enrichment was tested using the *z* statistic. The *z* score was converted to a *p* value and was FDR-adjusted in R environment.

### Comparison of proteome and phosphoproteome datasets

From the proteome data, those proteins that were 2.5 MAD away from the median (roughly equating to twofold change) in three out of four experiments were considered as regulated. For comparative analysis, proteins that displayed change in abundance at both phosphoproteome and proteome level, and those that displayed changes only in the phosphoproteome level were grouped into four distinct clusters. Functional enrichment for the cellular component was carried out for each of the clusters and the significantly enriched clusters (*p* value <0.05) in at least one of the groups were retained for hierarchical clustering and visualization as a heatmap. To perform gene-set enrichment analysis a pre-ranked list based on average fold change derived (ANXA1^-/-^/ANXA1^+/-^ ratio) from both proteome and phosphoproteome data was created. For genes with multiple phosphopeptides, fold changes corresponding to the most regulated phosphopeptides (highest or lowest fold change) were used for ranking. For the others that did not meet the previously set robust *z* score cutoff, those phosphopeptides with the highest ratio were used. Gene-set enrichment analysis (GSEA) was performed using all curated mouse GO biological processes and pathways individually for both the proteome and phosphoproteome data [33]. Statistical significance of the enrichment was assessed by permutation analysis, and mutually overlapping gene-set clusters with a *p* value <0.01 were visualized as a network using the Enrichment Map plugin as implemented in Cytoscape [34].

### Network analysis

Expression-driven transcription factor prediction analysis was carried out for all differentially regulated proteins in the proteome dataset using the Transcription Factor database (TRANSFAC) [35]. The analysis was performed against a background that included predictions for all proteins in our dataset and the significance of the enrichment was calculated using the one-sided Fisher’s exact test. The predicted transcription factors that were significantly enriched (*p* value <0.05) were visualized using the Treemap package from R. For construction of the interaction network we used protein-protein interactions curated in the InWeb_InBioMap database that has 2.8 times more interactions than other comparable resources and is also functionally superior [36]. The identified phosphoproteins along with the predicted transcription factors were mapped onto this network, and later filtered to include only edges connecting altered phosphoproteins or significant transcription factors or both. The phospho-network contained a total of 282 nodes and 779 edges. The network components were arranged into modules based on their functional categorization (such as GTPase or kinase), pathways or cellular localizations for better visualization. For automated cluster enrichment, the GLay community clustering algorithm was implemented and the clusters were extracted for visualization [37].

## Results

### Quantitative phosphoproteomics of ANXA1-deficient murine mammary gland cells

**Fig. 1:**
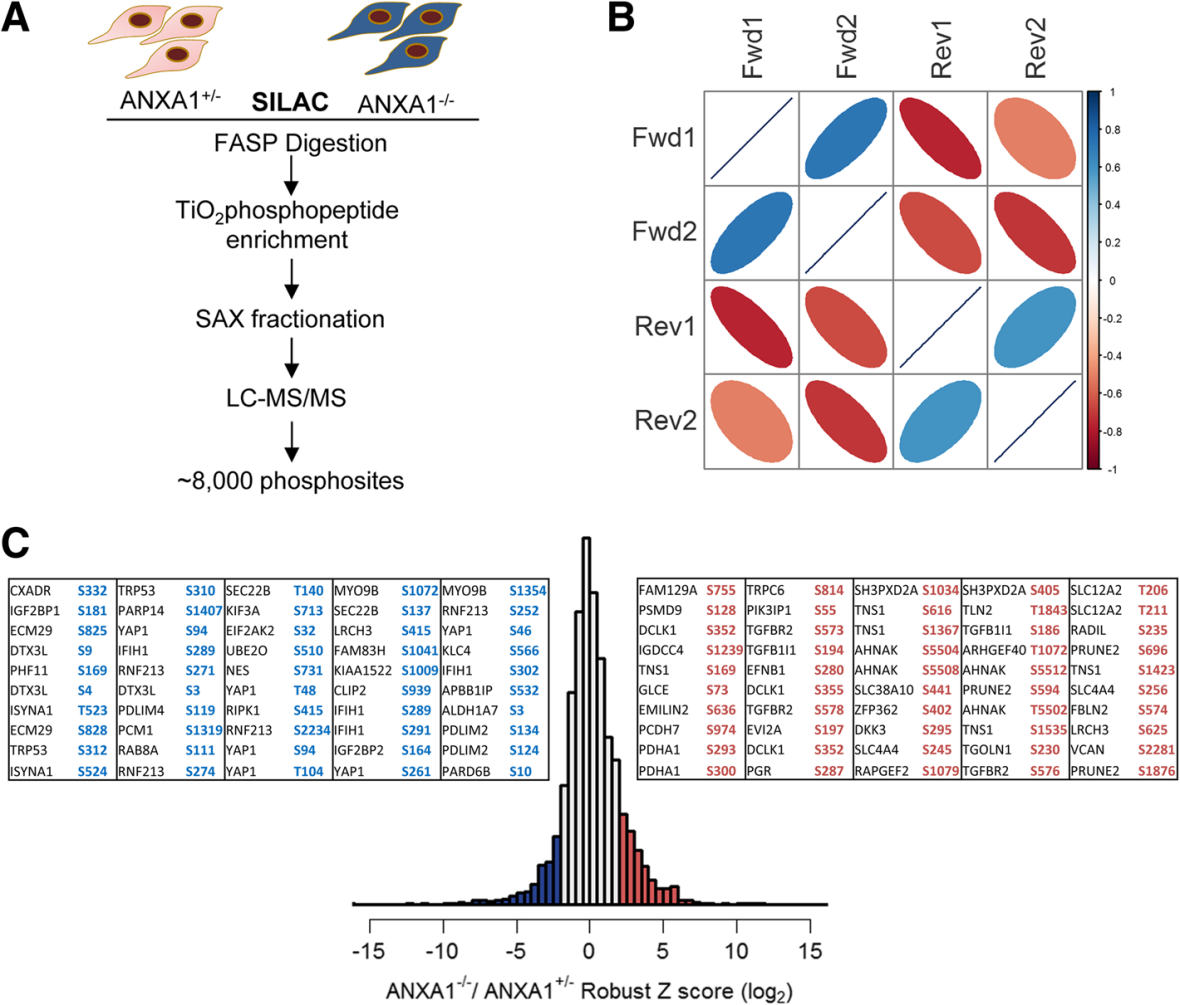
Phosphoproteome analysis of annexin-1 (ANXA1)-responsive phosphorylation changes. **a** Schematic representation of the phosphoproteome workflow used for the analysis of ANXA1^+/-^ and ANXA1^-/-^ mammary gland epithelial cells. The cells were stable isotope labeling with amino acids in cell culture (SILAC)-adapted and a total of four biological replicates including label reverse experiments were performed. **b** Correlation between the forward (Fwd) and reverse (Rev) experiments. Positive correlation between the forward and negative correlation between the label swap experiments can be observed. **c** Distribution of quantified phosphopeptides in one representative replicate. Those sites that were reliably regulated above or below the MAD threshold of ± 2 in three out of four replicates were considered ANXA1-responsive. The top 50 regulated sites with increased and decreased phosphorylation are shown. The robust *z* score is based on log_2_ normalized fold changes. FASP, filter-aided sample preparation; LC-MS/MS, liquid chromatography-tandem mass spectrometry; SAX, strong ion exchange

Our previous quantitative profiling of protein abundances in ANXA1^+/−^ and ANXA1^−/−^ murine mammary gland epithelial cells implicated a role for ANXA1 in breast cancer initiation and progression [21]. To further elucidate ANXA1-dependent signaling events, we performed SILAC-based quantitative phosphoproteome analysis of ANXA1^+/−^ and ANXA1^−/−^ murine mammary cells as depicted in Fig.1a and described in “Methods”. The collective analysis from biological duplicates of each forward (heavy ANXA1^-/-^ cells versus light ANXA1^+/-^ cells) and reverse experiments (heavy ANXA1^+/-^ cells versus light ANXA1^-/-^ cells) identified a total of 11,235 unique phosphopeptides carrying 8110 unique phosphorylation sites on 2626 proteins (Additional file 1: Table S1 and Additional file 2: Figure S1A). Of these, a majority (83%) was among the class I phosphorylation sites with high localization probability (≥0.75), and only 6465 sites (~80%) overlapped with previously annotated phosphorylation sites in the PhosphoSitePlus database (Additional file 2: Figure S1B). The total phosphosites identified in the individual biological replicates was comparable, and the replicates demonstrated good reproducibility and quantification accuracy as is evident from their correlation (Fig. 1b and Additional file 2: Figure S1C). While a majority of the phosphopeptides was singly phosphorylated (87%), about 11% displayed two phosphorylation sites per peptide and the rest harbored at least three sites.

Using a robust *z* score cutoff of 2 (median absolute deviation 2 above or below the median ratio roughly equating to 1.8-fold change) we identified 362 phosphosites on 224 proteins with increased phosphorylation and 220 sites on 153 proteins with decreased phosphorylation in three out of four experiments (Fig. 1c, Additional file 3: Figure S2 and Additional file 4: Table S2). Apart from these altered sites, 189 of these phosphoproteins also harbored additional sites that displayed no change in phosphorylation abundance, suggesting that these site-specific changes plausibly relay ANXA1-dependent signals in breast epithelial cells. Most of the regulated sites belonged to serine/threonine phosphorylation with only two tyrosine sites being regulated in Tensin-like C1 domain-containing phosphatase (TENC1) and cyclin-dependent kinase 1 (CDK1) (Additional file 3: Figure S2B). A few proteins including supervillin (SVIL), BAT2 domain-containing protein 1 (PRRC2C), MAP7 domain-containing 1 (MAP7D1) and leucine rich repeats and calponin homology domain containing 3 (LRCH3), however, displayed differential phosphorylation with both increased and decreased phosphorylation site abundance. These results clearly indicate that ANXA1 is extensively involved in protein phosphorylation signaling in breast epithelial cells.

Comparison of this phosphoproteome dataset to our previously reported proteome profiling (Additional file 5: Figure S3 and Additional file 6: Table S3) revealed that though 77% of the identified phosphorylation sites had a corresponding protein measure, a majority of the changes arose from site-specific differential phosphorylation and not due to changes in protein abundance. Interestingly, one of the proteins belonging to the antioxidant family, peroxiredoxin-6 (PRDX6), showed opposite regulation with elevated protein abundance and reduced phosphorylation at Thr44 spanning the catalytic center, suggesting possible enzymatic activity modulation by ANXA1.

### Functional portrait of ANXA1-regulated phosphoproteome

While the function of ANXA1 in mediating anti-inflammatory response is well-documented, a wide spectrum of cellular mechanisms regulated by ANXA1 has only been realized in recent years. In our study, we uncovered site-specific changes on several proteins including kinases and receptors reflecting the impact of ANXA1 on signaling pathways in mammary epithelial cells. Several mitogen-activated protein kinase (MAPK)-associated kinases such as MKNK2, MAP3K8 and RPS6KA3 and microtubule regulatory kinases such as DCLK1, MAST2 and MARK3 are among those with induced phosphorylation. This is interesting as some of these kinases have a profound influence on cell migration activities of pathways including WNT and NF-κB signaling. Enhanced phosphorylation of serine/threonine kinase receptor TGFBR2 on multiple serine sites at Ser573, Ser576 and Ser578 was also observed in ANXA1^-/-^ cells. This agrees with previous reports of ANXA1 regulating TGFβ signaling possibly via modulating receptor kinase activity [19]. Conversely, kinases involved in inflammatory response such as EIF2AK2 and RIPK1 displayed reduced phosphorylation. Decreased phosphorylations were also observed on kinases involved in cell adhesion and migration. For instance, the non-receptor tyrosine kinase ABL2 associated with actin filament organization and cytoskeletal remodeling displayed reduced phosphorylation on two serine sites flanking the F-actin binding region [38, 39]. For the receptor tyrosine kinase EPHA2, multiple sites displayed decreased phosphorylations including that on the functional site Ser898 that is implicated in promoting cell migration [40]. Among all the sites modulated in the absence of ANXA1, only a few are known regulatory sites (Additional file 7: Table S4), and the functional significance of the remaining are yet to be explored.

**Fig. 2:**
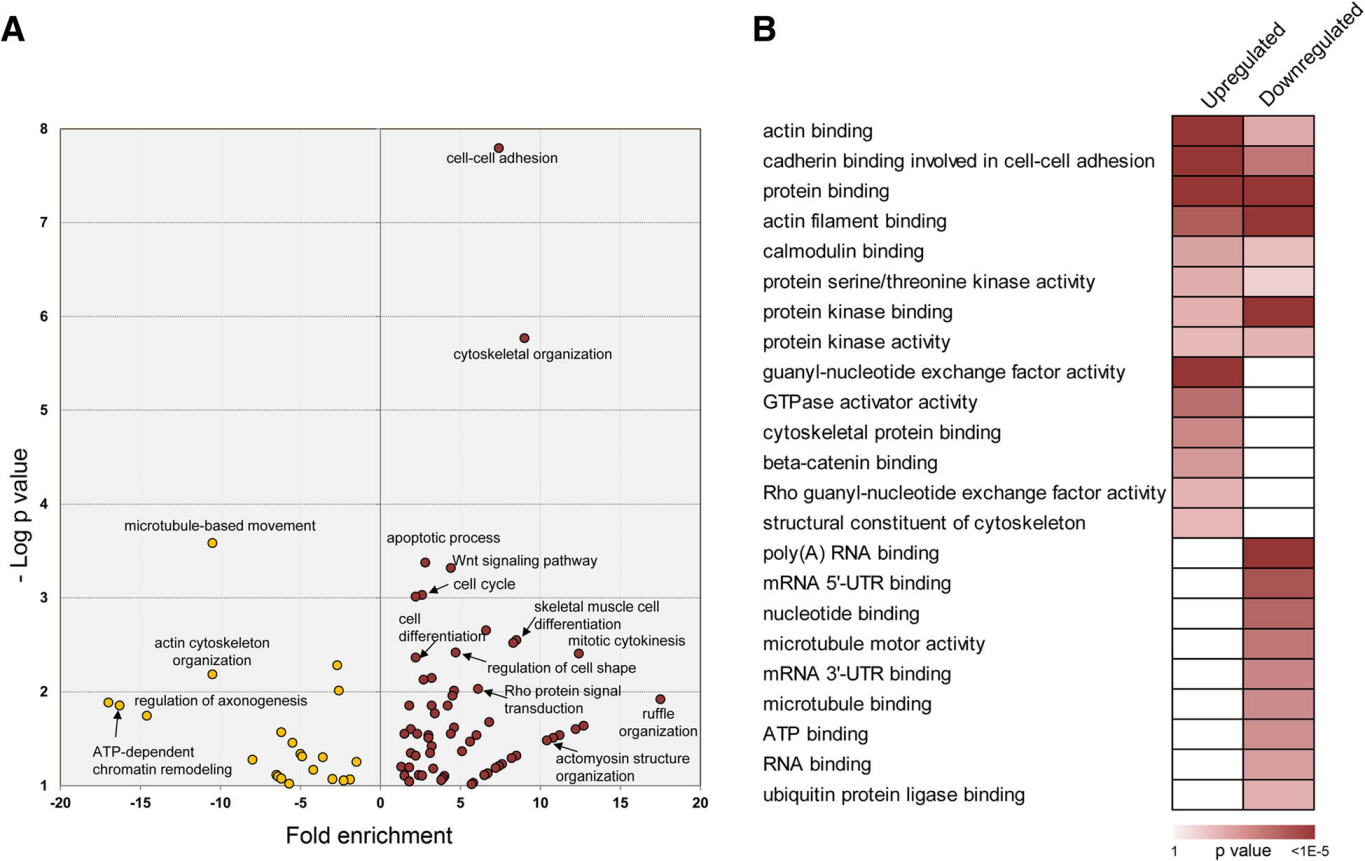
Functional enrichment of annexin-1 (ANXA1)-altered phosphoproteins. **a** Fold enrichment of ANXA1-responsive phosphoproteins for biological processes. Enrichment among the upregulated phosphoproteins are indicated by red and that among the downregulated phosphoproteins is indicated by yellow. **b** Enrichment of significantly enriched (*p* value <0.01) cellular components in either of the groups is visualized as a heatmap

Next, we looked into ontology-based enrichment for those modulated phosphoproteins (Fig. 2a). It became evident that a majority of the upregulated phosphoproteins were involved in cell-cell adhesion and cytoskeletal organization, particularly actin-filament-based processes. Accordingly, those components that govern cell shape and movement such as actomyosin structure, focal adhesions and stress fibers were selectively enriched (Additional file 8: Table S5). This is consistent with reports linking ANXA1 and cell migration, and underpins the role of ANXA1 in adhesion complexes. We also observed site-specific upregulation on proteins associated with the Rho family of GTPases that work as molecular switches in cell organization and movement. Specifically, several GTPase-activating protein (GAP) such as ARHGAP21, ARHGAP28 and ARHGAP29 that function as RhoA-GAP, and Rac-gunanine nucleotide exchange factor (GEF) DOCK7 displayed increase in phosphosite abundance. Our data also revealed an interesting link between ANXA1 and the WNT signaling pathway with several members including receptor LRP6, crosslinking factor MACF1 and antagonist dickkopf-3 (DKK3) showing increased phosphorylation in ANXA1-deficient mammary epithelial cells. LRP6 constitutes the WNT-ligand-induced co-receptor complex and confers active WNT signaling. We observed a reproducible increase in phosphorylation of two sites, Ser1490 and Thr1493, which is crucial for receptor activation and signal transduction [41], establishing that ANXA1 level may directly correlate with WNT pathway output. In concordance with these functions, the proteins with elevated site abundance also showed enhanced cadherin and β-catenin binding in comparison with those with reduced phosphorylation (Fig. 2b).

Proteins with downregulated sites in ANXA1-deficient mammary epithelial cells displayed preferential enrichment for microtubule-based processes and ATP-dependent chromatin remodeling. A few phosphoproteins with reduced site abundance were also enriched for actin cytoskeletal processes and localized to focal adhesions indicative of a complex network of phosphorylation-mediated events that establish cell movement. While the role of ANXA1 in regulating the actin cytoskeleton has been reported earlier, our dataset exposes its possible novel associations in influencing microtubule-filament-based processes. We uncovered reduced phosphosite abundance on microtubule motor proteins including kinesin and dynein, and on other microtubule-associated proteins such as MAP2 and CLIP2. It is worthy to note that one of the key proteins that influence microtubule stability, stathmin (STMN1) was found with increased phosphosite abundance at the functional site Ser16 that inhibits microtubule depolymerization in ANXA1-deficient cells, highlighting the pervasive role of ANXA1 in regulating microtubule dynamics [42].

**Fig. 3:**
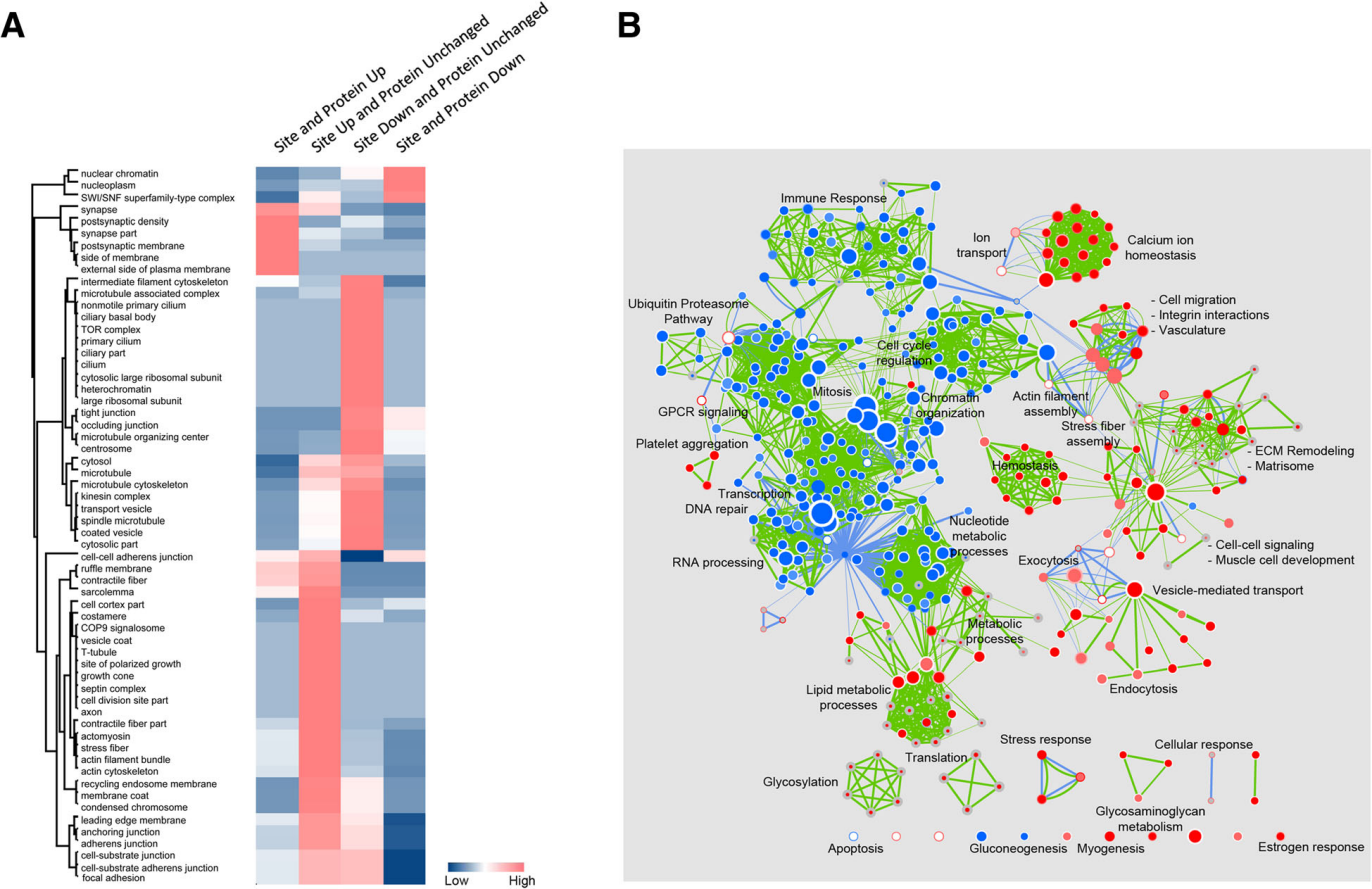
Comparative analysis of regulated proteins and phosphoproteins in annexin-1 (ANXA1)-deficient mammary epithelial cells. **a** Component enrichment of proteins that were categorized into those that demonstrated high protein and phosphosite abundance, high phosphosite abundance with no changes in protein level, low phosphosite abundance with no changes in protein level, and low phosphosite and protein abundance. The cellular localization significantly enriched in at least one of the four groups is depicted on the heatmap. High (pink) and low (blue) in the heat map represent statistical over-representation or under-representation, respectively. **b** Gene set enrichment analysis of pathways and biological processes performed individually for altered proteins and phosphoproteins that were overlaid together as an enrichment map. Functions significantly (*p* value <0.01) enriched in upregulated and downregulated proteins are represented by red and blue nodes, respectively. Processes involving phosphoproteins with increased or decreased site abundances are depicted as red and blue borders around the nodes. Those processes that are characterized only by phosphosite changes have blue edges while the pathways and processes that are commonly enriched in both have green edges

Next, we asked how the functions of those proteins that changed in total or site abundance compared with each other in ANXA1-deficient mammary cells (Additional file 3: Figure S2A, Additional file 9: Figure S4 and Additional file 10: Table S6). For this purpose, we first analyzed the cell component distribution of protein subsets that were regulated at both the proteome and phosphoproteome level or only at the phosphoproteome level by performing combined hierarchical clustering (Additional file 11: Table S7). We observed distinct clusters specific to each subset (Fig. 3a). For example, a subset of proteins that localized to the exterior plasma membrane and ruffle structures displayed elevated protein and phosphosite abundance. The proteins that exclusively displayed elevated phosphorylation with no change at protein level traversed a wide range of cellular components, particularly spanning all the junctional and focal adhesion components. Phosphoproteins were specifically enriched at the leading edge membrane that constitutes the lamellipodium, stress fiber, actomyosin and contractile fibers, revealing extensive phosphosignaling and rewiring of the cell adhesion machinery upon loss of ANXA1. Apart from these, proteins that localized to endosome membrane displayed only phosphosite upregulation. On the other hand, proteins that constitute the microtubule cytoskeleton, spindle and kinesin complex displayed only reduced phosphosite abundance with no protein level changes. Only a small subset of proteins showed decrease in both phosphorylation and corresponding protein level, and these localized to chromatin remodeling components.

In addition, by performing GSEA of canonical biological pathways and processes [33] individually for both the ranked proteome and phosphosite abundance, we showed distinct functions fine-tuned by the protein subsets (Fig. 3b). From the enrichment map it is evident that processes representing immune response, nucleotide metabolic process and cell cycle regulation are majorly represented by downregulated proteins. Interestingly, the proteins associated with cell migration, integrin-mediated adhesion, extracellular matrix (matrisome), stress and estrogen response are enriched in the subset with upregulation of both protein and phosphosite abundance. Proteins involved in calcium ion homeostasis also displayed similar trends underscoring the crucial role of ANXA1 in regulating calcium dynamics and associated signaling. Again, this analysis also highlighted that stress fiber and actin filament assembly along with exocytosis events are exclusively driven by proteins with increased phosphorylation.

Altogether, the current dataset suggests that ANXA1 is crucially involved in reshaping the cytoskeleton and modulates cell adhesion or migratory properties by fine-tuning both protein and phosphorylation levels that work in concert with each other.

### Kinases regulated by ANXA1 in mammary epithelial cells

**Fig. 4:**
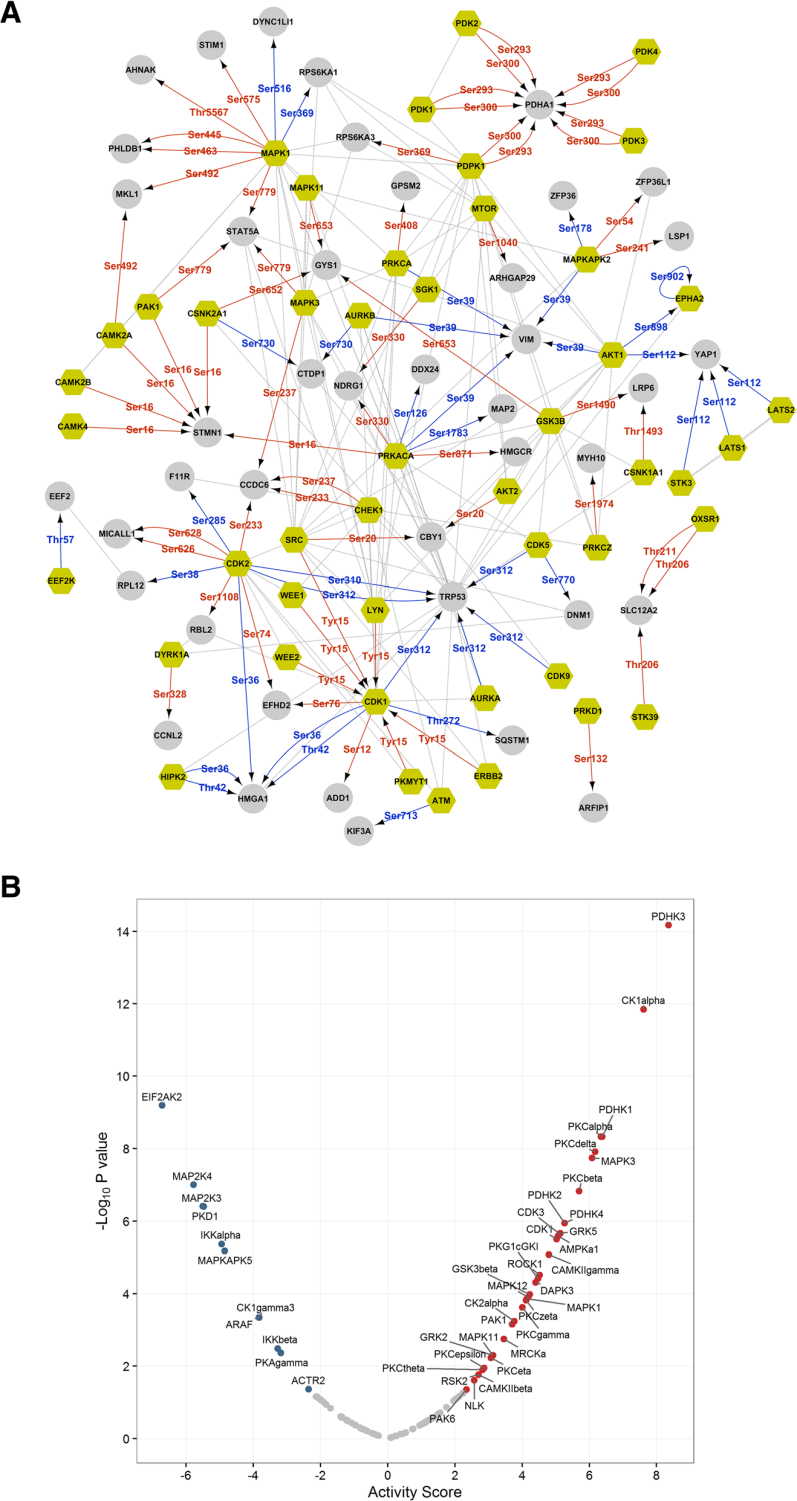
Annexin-1 (ANXA1)-altered kinase profiles. **a** Experimentally curated kinase-substrate relationships for ANXA1-responsive phosphoproteins are visualized as a kinome network. The specific sites that are phosphorylated by the kinase are shown as red (increased abundance) or blue (decreased abundance) edges. High-scoring protein-protein interactions are additionally mapped onto the network. Yellow nodes represent all kinases. **b** Kinase-substrate enrichment analysis was carried out for predicted upstream kinases. Positive activity score indicates higher activity and negative scores correspond to lower activity. Those kinases enriched with *p* value <0.05 are indicated with red and blue dots.

We next mapped site-specific information of the phosphoproteins against a comprehensive knowledge base of experimentally derived kinase-substrate relationships (KSR). Of all the regulated sites, upstream kinase was experimentally curated only for 56 phosphosites on 45 proteins. The interconnectivity of the kinome network (Fig. 4a) was obtained through mapping of protein-protein interactions. With many substrates of MAPK1 showing increased phosphorylation in the network, it is evident that MAPK1 displays elevated activity, and similarly, AKT1 shows reduced activity upon ANXA1 knockout. Whether such modulations are a direct effect of ANXA1 in breast epithelial cells or arise from feedback regulations between the MAPK/ERK and PI3K/AKT pathways is still not known [43–45]. While for these and some other kinases such as PAK1 and Aurora B such activity prediction was possible from the network, for a few others including PKA and CDK the substrates displayed both increased and decreased phosphorylation. This suggests that the activities of these kinases may not be turned on/off entirely, but possibly rewired wherein some substrates are selectively phosphorylated while others are not.

Though the kinome network highlighted KSRs from diverse kinase families, it is not representative of the entire signaling network as many of our regulated phosphosites did not have experimentally curated upstream kinase. Hence, we additionally predicted KSRs for all regulated phosphoproteins using the NetworKIN algorithm that assigns kinases based on linear consensus motifs and network proximity [31]. We performed kinase-substrate enrichment analysis (KSEA) to derive significantly modulated kinase along with their predicted activity scores (Fig. 4b). This analysis mirrored some of the findings from the previous kinome network including activation of MAPK1, CAMK2, CK2A, and highlighted other kinases that could be perturbed in the ANXA1 signaling network. We observed CDK family kinases CDK1 and CDK3 to be activated upon loss of ANXA1, and this along with enhanced MAPK1/3 activity possibly constitutes a pro-proliferative signal. Strikingly, kinases such as PAK1, ROCK1 and MRCK were found to be activated upon ANXA1 knockout. These kinases play important roles in determining cell properties during migration, and thus their activation focuses on the rewiring of adhesion network upon loss of ANXA1. Also, a subset of the kinases was predicted to be deactivated in ANXA1-deficient mammary cells, and many of these were associated with cellular stress and cytokine response. These included kinases IKKα and IKKβ that are activators of NF-κB transcription factor. Notably, ANXA1 has been previously reported to constitutively activate NF-κB in breast cancer cells via the IKK complex [46], and our phosphoproteome data also predicts such a positive correlation between ANXA1 and NF-κB activity status.

### Mechanistic network of ANXA1-regulated phosphoproteome

**Fig. 5:**
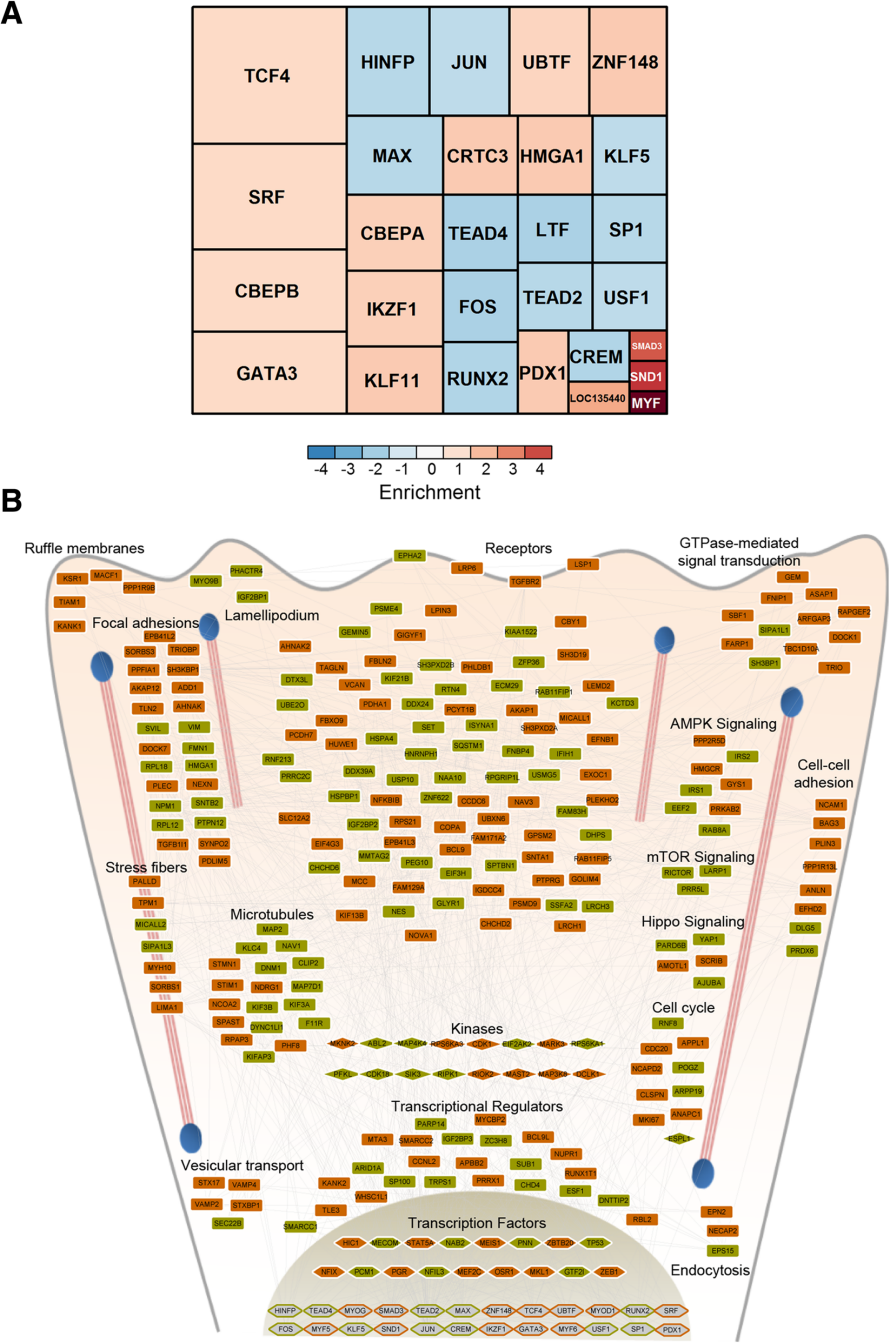
Annexin-1 (ANXA1)-regulated phosphoproteome network. **a** Transcription factors predicted to be enriched among the upregulated and downregulated proteins are visualized as a treemap. The size of the squares indicates the number of transcriptional targets and the color of the squares represent enrichment factor. Only significantly enriched (*p* value <0.05) transcription factors are shown. **b** Schematic network of the ANXA1-regulated phosphoproteome interacting with the predicted transcription factors. Proteins were grouped into their respective functional process or localizations for visualization purpose. The stress fibers are depicted as pink lines and the focal adhesions are marked as blue ovals. ANXA1-responsive phosphoproteins with increased site abundance are shown with brown-filled nodes and those with decreased site abundance are represented by green-filled nodes. Diamond-shaped nodes represent kinases. Nodes with only border colors denote predicted upregulated (brown) and downregulated (green) transcription factors

To map the ANXA1-modulated network to its entirety, it is necessary to understand where the downstream signaling impinges and which transcription factors are affected as a consequence. We used changes in protein abundance measures from our previous study to predict transcription factor activation status in ANXA1-deficient mammary cells. Using one-sided Fisher’s exact test, we derived a set of 28 transcription factors (*p* value <0.05) that were potentially regulated in the absence of ANXA1 (Fig. 5a). We found that the targets of cellular stress-associated transcription factors FOS and JUN were specifically enriched among the downregulated proteins. This aligns with our kinase analysis, which predicted reduced activity for kinase MAP2K4 that lies upstream of JUN in the same pathway. Transcriptional targets of TEA domain family (TEAD) transcription factors that are associated with YAP1/TAZ from the Hippo signaling pathway [47], also showed specific enrichment among the downregulated proteins. Such a link between ANXA1 and the Hippo signaling pathway has not been established previously to our knowledge. Other functional partners of YAP1 including RUNX2 also showed negative enrichment [48]. Alternatively, the activities of myogenic regulatory factor (MYF) and serum response factor (SRF) were found to negatively correlate with ANXA1 abundance. These factors are crucial for actin-cytoskeletal organization and focal adhesion assembly [49], and thus correlate with enrichment observed based on phosphoproteome data. While some of the kinases and transcription factors we predicted through analyses of our phosphoproteome and proteome dataset have previously been associated with ANXA1, several novel associations and pathways became evident through our in-depth investigation.

We next combined the predicted transcription factors along with the regulated phosphoproteins, and assessed the functional connectivity among them. We curated protein-protein interactions from InWeb_InBioMap [36], a large data compendium for high quality protein-protein interaction network, and built an interaction network consisting of 282 proteins including 258 differentially regulated phosphoproteins. A contextual view of the network is shown in Fig. 5b with proteins grouped into distinct clusters based on their overrepresented pathways/processes. We observed that many regulated phosphoproteins displayed direct interactions with several predicted transcription factors. A majority of these interactions were mediated by transcriptional regulators that were differentially phosphorylated in ANXA1-deficient cells. The precise nature of these interactions, however, and whether the phosphorylation status may have any influence on them is not known for most of them.

The network revealed that a majority of the proteins in focal adhesion, stress fibers and ruffled membranes displayed increased phosphorylation and as expected also displayed high connectivity among one another (Additional file 12: Table S8). In fact a cluster assessment based on GLay community clustering also highlighted cell adhesion as one of the top enriched clusters in the network highlighting the influence of ANXA1 in governing adhesion dynamics (Additional file 13: Figure S5) [37]. Projections that form on the leading edge, the lamellipodium, constituted proteins that displayed reduced phosphorylation. We found the adaptor protein talin (TLN2) that is crucial for focal adhesion initiation with elevated phosphorylation at Thr1843 upon ANXA1 loss. Talin establishes core cell extracellular matrix (ECM) links by associating and activating integrin, and simultaneously binding to actin and other cytoskeletal proteins [50]. Adhesion life cycle encompasses recruitment of various proteins at different stages, and only a fraction of adhesions survive to become large stable focal adhesion. Finally, disassembly of these adhesions occurs to enable cell spreading and forward movement. This disassembly and destabilization can be microtubule-mediated, wherein microtubules either stimulate ABL2 to inhibit RhoA, which is crucial for adhesion maturation and stress fiber formation or simply their depolymerized state can induce adhesion instability [51–53]. We observed many phosphorylation changes on microtubule-associated proteins including the increased phosphorylation on stathmin that inhibits microtubule depolymerization/catastrophe [42]. This along with reduced phosphorylation on ABL2 kinase possibly points to defects in microtubule-mediated adhesion disassembly, thus favoring enhanced adhesion and less migratory capacity of ANXA1-deficient mammary epithelial cells.

Apart from these major structural components, other connected proteins mainly belonged to cell signaling and cell-cycle-related processes (Additional file 12: Table S8). It is evident from the network that many of these regulated proteins also share interactions with components in focal adhesion and stress fibers underscoring complex interplay among ANXA1-regulated network. Notably, we observed Rictor, which is a component of mTOR complex 2 with reduced phosphorylation on Thr1592. mTORC2 is a regulator of cytoskeletal dynamics and stimulates Rho GTPases and F-actin stress fibers [54]. The fact that many of the regulated phosphoproteins converge on these structural assemblies emphasizes the notion that ANXA1 primarily governs cell adhesion and migratory properties in mammary epithelial cells.

## Discussion

The spectrum of functions attributed to ANXA1 has expanded in recent years from an anti-inflammatory protein to that of a regulator for several cellular processes including proliferation, apoptosis and migration. With several reports suggesting correlation between ANXA1 and breast cancer outcome, we earlier focused on characterizing ANXA1-responsive changes in global proteome during breast cancer initiation and breast cancer tumorigenesis [21, 22]. The repertoire of proteins that were modulated in normal breast epithelial cells and tumor cells in response to ANXA1 deficiency was distinct. Yet, both the studies emphasized a strong link between ANXA1 and migratory properties of breast cancer cells. Thus following our previous work, in this study we have taken a step further to systematically analyze ANXA1-responsive changes in the phosphorylation profile of mammary gland cells derived from ANXA-1 deficient mice to reveal signaling networks and complexes impacted by ANXA1 and thus understand its role in breast cancer initiation.

Through our combined analysis of the proteome and phosphoproteome datasets we observed functionally distinct fine-tuning of several processes. Collectively, many of the proteins involved in cytoskeletal organization displayed changes either in total abundance, phosphosite abundance or both. However, unlike in the proteome dataset wherein we observed enrichment of the DNA damage response pathway among the downregulated proteins, there was no specific enrichment for this pathway among the regulated phosphoproteins. Similarly, many of the inflammatory-response-associated proteins majorly displayed changes in protein abundance only. This suggested that ANXA1-responsive translational and post-translational mechanisms span discrete functional processes, but for mechanisms involving ECM remodeling and adhesion complexes they work in concert to dictate cell motility fate.

We observed several cell adhesion and migration-associated proteins among the ANXA1-responsive phosphoproteins (Additional file 14: Table S9). Of note, our previous study established that ANXA1-deficient mammary epithelial cells are less migratory as compared with ANXA1^+/−^ cells [21]. Accordingly, we found several proteins that can function as a part of the adhesome complex to have increased phosphorylation upon ANXA1-deficiency. For instance, several proteins including PDLIM5, vinexin, ponsin, palladin and supervillain, which constitute core cell adhesion machinery, and hence part of the consensus adhesome [55], displayed changes in phosphorylation in response to ANXA1. Some of the phosphorylation we observed on these proteins including those on adaptor protein vinexin (Ser412 mouse/Ser348 human) have been previously reported as adhesion-complex-specific phosphorylation sites [56]. While the upregulation of several of these regulated phosphoproteins are known to induce formation of focal adhesion and stress fiber, the precise roles of phosphorylation on these proteins still remain unclear. Also, we observed several GAPs and GEFs with modulated phosphorylation. With predicted activity changes for PAK and ROCK1 kinases that occur downstream of Rac1 and RhoA, respectively, we posit ANXA1-mediated crosstalk between Rho and Rac GTPases that possibly drives cellular decisions on adhesion and migration. Though tyrosine phosphorylation events on adhesion proteins such as focal adhesion kinase (FAK), Src, paxillin etc. are characteristic of adhesion and integrin signaling, we did not detect these tyrosine sites in our data. This may be partly because we used whole cell lysate and not specifically isolated adhesion complexes to perform our phosphoproteome profiling, and also that we did not perform any phosphotyrosine enrichment in this study. However, it is worthy to stress that the importance of several serine/threonine phosphorylation events by far remains underappreciated in the context of adhesion signaling.

Microtubule dynamics are a key factor in controlling cell cycle and other processes such as intracellular trafficking, signaling and events leading to cell migration [57, 58]. Through coordinated polymerization and depolymerization, the spatial and temporal organization of the microtubule network largely governs directionality and also restrains cell movement. We uncovered several phosphorylation changes on microtubule associated proteins and those that regulate its dynamics in ANXA1-deficient cells. Such a role for ANXA1 in regulating microtubule dynamics has not been established earlier to our knowledge. One of the major regulators of microtubule dynamics, stathmin, was found with elevated phosphorylation on Ser16 in ANXA1-deficient cells. The cell cycle-regulated stathmin promotes disassembly or destabilization of microtubules, and hence is turned off at the onset of mitosis to allow assembly of mitotic spindle [59]. The “on” and “off” status of stathmin is mainly regulated by phosphorylation at Ser16 with increase in phosphorylation inhibiting its destabilizing activity [42, 60]. Thus, in ANXA1-deficient mammary cells, we predict a decrease in microtubule depolymerization. Indeed, this process also affects disassembly of focal adhesion, and we attribute the observed increase in adhesion to such defects as well. Of note, low levels of Ser16-phosphorylated stathmin correlate with metastatic states of breast cancer [61], and an increase in Ser16 has been shown to reduce migration in esophageal cancer cells [62], thus underscoring its influence in limiting migration and possibly increasing adhesion. Interestingly, our kinase analysis also predicted higher activity for CAMK2, PAK and RSK2 kinases that are responsible for its phosphorylation.

Our phosphoproteome data also highlighted altered phosphorylation on proteins involved in signaling pathways including the WNT, Hippo and AMPK cascades. AMPK activation was also predicted by upstream kinase analysis, and this negative correlation was consistent with previous reports wherein ANXA1 knockdown in breast cancer cells activated AMPK [63]. We particularly observed modulation of Hippo signaling and reduced transcriptional output via TEAD transcription factors. Loss of adherens junction deregulates Hippo signaling and hyperactivates its downstream effectors YAP/TAZ [64]. We speculate on adhesion-induced modulation of Hippo signaling pathway in ANXA1-deficient mammary cells, wherein YAP protein levels and activity are suppressed (YAP1 levels are indeed reduced upon ANXA1 knockout [21]) leading to reduced activation of TEAD transcription factors. Notably, the phosphorylation of a basolateral component SCRIB, the loss of which activates YAP/TAZ [64], also displayed elevated phosphorylation in ANXA1-deficient cells. Thus we posit there is correlation between ANXA1 and Hippo signaling outcome and hypothesize that high levels of ANXA1 could hyperactivate YAP/TAZ leading to increased TEAD transcriptional output.

## Conclusions

Collectively, this study expands our understanding of the molecular mechanisms modulated by ANXA1 in mammary epithelial cells by an in-depth investigation of the ANXA1-responsive signaling network. Our data highlight reorganization of cellular structural components and rewiring of signaling networks upon loss of ANXA1 that partly explain the broad spectrum of roles proposed for ANXA1 in breast cancer.

## Additional files

Additional file 1: Table S1. List of all identified phosphorylation sites in mammary epithelial cells from ANXA1-heterozygous and ANXA1-deficient mice. (XLSX 10173 kb)

Additional file 2: Figure S1. Phosphoproteomic data quality and reproducibility. **A** Distribution of Andromeda scores of the identified phosphorylated peptides show that most peptides were identified with high scores with a median score of 130. **B** Distribution of localization probabilities of identified phosphorylation sites. The median probability was ~ 0.97. **C** Assessment of reproducibility between the four biological replicates carried out with SILAC label swapping shows optimal reproducibility among the forward and reverse experiments. The correlation scores are indicated in each scatter plot. (PDF 1803 kb)

Additional file 3: Figure S2. ANXA1-regulated phosphoproteome in mammary epithelial cells. **A** Distribution of robust *z* scores of the quantified phosphopeptides. The regions highlighted in red and blue correspond to upregulated and downregulated phosphopeptides, respectively. The sites that were reliably regulated above or below the MAD threshold of ± 2 in three out of four replicates were considered ANXA1-responsive and these at least displayed a 1.8-fold change. The robust *z* scores were based on log_2_ normalized fold changes. **B** Distribution of serine, threonine and tyrosine sites among the regulated phosphorylation sites. (PDF 1059 kb)

Additional file 4: Table S2. List of ANXA1-responsive phosphorylation sites in mammary epithelial cells from ANXA1-heterozygous and ANXA1-deficient mice. (XLSX 1001 kb)

Additional file 5: Figure S3. Comparison of quantified proteome and phosphoproteome in ANXA1-deficient mammary epithelial cells. **A** Number of class I phosphorylation sites with corresponding protein quantification. Except for 1550 sites on 765 proteins that had no corresponding protein measure, the rest of the sites mapped to 1765 proteins with abundance measures. **B** Intensity-based density plot comparing protein and phosphorylation abundance shows poor correlation. (PDF 1234 kb)

Additional file 6: Table S3. List of all identified proteins in mammary epithelial cells from ANXA1-heterozygous and ANXA1-deficient mice. (XLSX 11902 kb)

Additional file 7: Table S4. Site-specific functions of ANXA1-regulated phosphorylation sites. (XLSX 23 kb)

Additional file 8: Table S5. Cellular component enrichment of ANXA1-modulated phosphoproteins. (XLSX 10 kb)

Additional file 9: Figure S4. ANXA1-regulated proteins in mammary epithelial cells. Distribution of robust *z* scores of the quantified proteins. The regions highlighted in orange and blue correspond to upregulated and downregulated phosphopeptides, respectively. Only those proteins regulated in at least three out of four experiments were considered regulated. (PDF 786 kb)

Additional file 10: Table S6. List of all ANXA1-regulated proteins in mammary epithelial cells from ANXA1-heterozygous and ANXA1-deficient mice. (XLSX 819 kb)

Additional file 11: Table S7. Enrichment of cellular component terms among the different categories. (XLSX 12 kb)

Additional file 12: Table S8. Summary of associated pathways and localizations of ANXA1-responsive phosphoproteins. (XLSX 29 kb)

Additional file 13: Figure S5. Clusters enriched in ANXA1-regulated protein interaction network. Integrated protein-protein interaction network was constructed using those proteins with ANXA1-responsive phosphorylation changes along with transcription factors predicted from ANXA1-regulated proteome. Clusters were identified using GLay community structure detection and the top clusters identified along with their associated functions are shown. (PDF 5678 kb)

Additional file 14: Table S9. Migration-associated ANXA1-responsive phosphoproteins. (XLSX 43 kb)

## Abbreviations

ANXA1: Annexin-1
CAMK2: Calmodulin-dependent protein kinase II
CDK1: Cyclin-dependent kinase 1
DKK3: Dickkopf-3
ECM: Extracellular matrix
EMT: Epithelial-mesenchymal transition
ERK: Extracellular signal-regulated kinase
FAK: Focal adhesion kinase
FASP: Filter-aided sample preparation
FDR: False discovery rate
GAP: GTPase-activating protein
GEF: Gunanine nucleotide exchange factor
GO: Gene Ontology
GSEA: Gene-set enrichment analysis
HPRD: Human Protein Reference Database
IKK: IκB kinase
kDa: kiloDalton
KSEA: Kinase-substrate enrichment analysis
KSR: Kinase-substrate relationships
LRCH3: Leucine rich repeats and calponin homology domain containing 3
MAD: Median absolute deviation
MAP7D1: MAP7 domain containing 1
MAPK: Mitogen-activated protein kinase
MYF: Myogenic regulatory factor
PAK: P21 activated kinase
PEG: poly(ethylene) glycol
PRDX6: Peroxiredoxin-6
PRRC2C: BAT2 domain-containing protein 1
SILAC: Stable isotope labeling with amino acids in cell culture
SRF: Serum response factor
STMN1: Stathmin
SVIL: Supervillin
TEAD: TEA domain family
TENC1: Tensin-like C1 domain-containing phosphatase
TFA: Trifluoroacetic acid
TGF: Transforming growth factor
TLN2: Talin 2

## References

[1] Flower RJ, Rothwell NJ (1994). Lipocortin-1: cellular mechanisms and clinical relevance. Trends Pharmacol Sci.

[2] Lim LH, Pervaiz S (2007). Annexin 1: the new face of an old molecule. Faseb J.

[3] Perretti M, D'Acquisto F (2009). Annexin A1 and glucocorticoids as effectors of the resolution of inflammation. Nat Rev Immunol.

[4] Scannell M, Flanagan MB, de Stefani A, Wynne KJ, Cagney G, Godson C (2007). Annexin-1 and peptide derivatives are released by apoptotic cells and stimulate phagocytosis of apoptotic neutrophils by macrophages. J Immunol.

[5] Sugimoto MA, Vago JP, Teixeira MM, Sousa LP (2016). Annexin A1 and the resolution of inflammation: modulation of neutrophil recruitment, apoptosis, and clearance. J Immunol Res.

[6] D'Acquisto F, Perretti M, Flower RJ (2008). Annexin-A1: a pivotal regulator of the innate and adaptive immune systems. Br J Pharmacol.

[7] D'Acquisto F, Paschalidis N, Sampaio AL, Merghani A, Flower RJ, Perretti M (2007). Impaired T cell activation and increased Th2 lineage commitment in Annexin-1-deficient T cells. Eur J Immunol.

[8] Yang YH, Song W, Deane JA, Kao W, Ooi JD, Ngo D (2013). Deficiency of annexin A1 in CD4+ T cells exacerbates T cell-dependent inflammation. J Immunol.

[9] Grewal T, Enrich C (2009). Annexins–modulators of EGF receptor signalling and trafficking. Cell Signal.

[10] Alldridge LC, Harris HJ, Plevin R, Hannon R, Bryant CE (1999). The annexin protein lipocortin 1 regulates the MAPK/ERK pathway. J Biol Chem.

[11] Liu YF, Zhang PF, Li MY, Li QQ, Chen ZC (2011). Identification of annexin A1 as a proinvasive and prognostic factor for lung adenocarcinoma. Clin Exp Metastasis.

[12] Bai XF, Ni XG, Zhao P, Liu SM, Wang HX, Guo B (2004). Overexpression of annexin 1 in pancreatic cancer and its clinical significance. World J Gastroenterol.

[13] Lin Y, Lin G, Fang W, Zhu H, Chu K (2014). Increased expression of annexin A1 predicts poor prognosis in human hepatocellular carcinoma and enhances cell malignant phenotype. Med Oncol.

[14] Geary LA, Nash KA, Adisetiyo H, Liang M, Liao CP, Jeong JH (2014). CAF-secreted annexin A1 induces prostate cancer cells to gain stem cell-like features. Mol Cancer Res.

[15] Yang Y, Liu Y, Yao X, Ping Y, Jiang T, Liu Q (2011). Annexin 1 released by necrotic human glioblastoma cells stimulates tumor cell growth through the formyl peptide receptor 1. Am J Pathol.

[16] Wang LP, Bi J, Yao C, Xu XD, Li XX, Wang SM (2010). Annexin A1 expression and its prognostic significance in human breast cancer. Neoplasma.

[17] Yom CK, Han W, Kim SW, Kim HS, Shin HC, Chang JN (2011). Clinical significance of annexin A1 expression in breast cancer. J Breast Cancer.

[18] Sobral-Leite M, Wesseling J, Smit VT, Nevanlinna H, van Miltenburg MH, Sanders J (2015). Annexin A1 expression in a pooled breast cancer series: association with tumor subtypes and prognosis. BMC Med.

[19] de Graauw M, van Miltenburg MH, Schmidt MK, Pont C, Lalai R, Kartopawiro J (2010). Annexin A1 regulates TGF-beta signaling and promotes metastasis formation of basal-like breast cancer cells. Proc Natl Acad Sci U S A.

[20] Parsons JT, Horwitz AR, Schwartz MA (2010). Cell adhesion: integrating cytoskeletal dynamics and cellular tension. Nat Rev Mol Cell Biol.

[21] Swa HL, Blackstock WP, Lim LH, Gunaratne J (2012). Quantitative proteomics profiling of murine mammary gland cells unravels impact of annexin-1 on DNA damage response, cell adhesion, and migration. Mol Cell Proteomics.

[22] Swa HL, Shaik AA, Lim LH, Gunaratne J (2015). Mass spectrometry based quantitative proteomics and integrative network analysis accentuates modulating roles of annexin-1 in mammary tumorigenesis. Proteomics.

[23] Wiśniewski JR, Nagaraj N, Zougman A, Gnad F, Mann M (2010). Brain phosphoproteome obtained by a FASP-based method reveals plasma membrane protein topology. J Proteome Res.

[24] Wiśniewski JR, Zougman A, Mann M (2009). Combination of FASP and stagetip-based fractionation allows in-depth analysis of the hippocampal membrane proteome. J Proteome Res.

[25] Cox J, Mann M (2008). MaxQuant enables high peptide identification rates, individualized p.p.b.-range mass accuracies and proteome-wide protein quantification. Nat Biotechnol.

[26] Cox J, Matic I, Hilger M, Nagaraj N, Selbach M, Olsen JV (2009). A practical guide to the MaxQuant computational platform for SILAC-based quantitative proteomics. Nat Protoc.

[27] Huang DW, Sherman BT, Tan Q, Kir J, Liu D, Bryant D (2007). DAVID Bioinformatics Resources: expanded annotation database and novel algorithms to better extract biology from large gene lists. Nucleic Acids Res.

[28] Domanova W, Krycer J, Chaudhuri R, Yang P, Vafaee F, Fazakerley D (2016). Unraveling kinase activation dynamics using kinase-substrate relationships from temporal large-scale phosphoproteomics studies. PLoS One.

[29] Szklarczyk D, Franceschini A, Wyder S, Forslund K, Heller D, Huerta-Cepas J (2015). STRING v10: protein-protein interaction networks, integrated over the tree of life. Nucleic Acids Res.

[30] Shannon P, Markiel A, Ozier O, Baliga NS, Wang JT, Ramage D (2003). Cytoscape: a software environment for integrated models of biomolecular interaction networks. Genome Res.

[31] Horn H, Schoof EM, Kim J, Robin X, Miller ML, Diella F (2014). KinomeXplorer: an integrated platform for kinome biology studies. Nat Methods.

[32] Casado P, Rodriguez-Prados JC, Cosulich SC, Guichard S, Vanhaesebroeck B, Joel S (2013). Kinase-substrate enrichment analysis provides insights into the heterogeneity of signaling pathway activation in leukemia cells. Sci Signal.

[33] Subramanian A, Tamayo P, Mootha VK, Mukherjee S, Ebert BL, Gillette MA (2005). Gene set enrichment analysis: a knowledge-based approach for interpreting genome-wide expression profiles. Proc Natl Acad Sci U S A.

[34] Isserlin R, Merico D, Voisin V, Bader GD (2014). Enrichment map - a cytoscape app to visualize and explore OMICs pathway enrichment results. F1000Res.

[35] Matys V, Fricke E, Geffers R, Gossling E, Haubrock M, Hehl R (2003). TRANSFAC: transcriptional regulation, from patterns to profiles. Nucleic Acids Res.

[36] Li T, Wernersson R, Hansen RB, Horn H, Mercer J, Slodkowicz G (2017). A scored human protein-protein interaction network to catalyze genomic interpretation. Nat Methods.

[37] Su G, Kuchinsky A, Morris JH, States DJ, Meng F (2010). GLay: community structure analysis of biological networks. Bioinformatics.

[38] Kain KH, Klemke RL (2001). Inhibition of cell migration by Abl family tyrosine kinases through uncoupling of Crk-CAS complexes. J Biol Chem.

[39] Wang Y, Miller AL, Mooseker MS, Koleske AJ (2001). The Abl-related gene (Arg) nonreceptor tyrosine kinase uses two F-actin-binding domains to bundle F-actin. Proc Natl Acad Sci U S A.

[40] Miao H, Li DQ, Mukherjee A, Guo H, Petty A, Cutter J (2009). EphA2 mediates ligand-dependent inhibition and ligand-independent promotion of cell migration and invasion via a reciprocal regulatory loop with Akt. Cancer Cell.

[41] Zeng X, Tamai K, Doble B, Li S, Huang H, Habas R (2005). A dual-kinase mechanism for Wnt co-receptor phosphorylation and activation. Nature.

[42] Wittmann T, Bokoch GM, Waterman-Storer CM (2004). Regulation of microtubule destabilizing activity of Op18/stathmin downstream of Rac1. J Biol Chem.

[43] Mendoza MC, Er EE, Blenis J (2011). The Ras-ERK and PI3K-mTOR pathways: cross-talk and compensation. Trends Biochem Sci.

[44] Yu CF, Liu ZX, Cantley LG (2002). ERK negatively regulates the epidermal growth factor-mediated interaction of Gab1 and the phosphatidylinositol 3-kinase. J Biol Chem.

[45] Aksamitiene E, Kiyatkin A, Kholodenko BN (2012). Cross-talk between mitogenic Ras/MAPK and survival PI3K/Akt pathways: a fine balance. Biochem Soc Trans.

[46] Bist P, Leow SC, Phua QH, Shu S, Zhuang Q, Loh WT (2011). Annexin-1 interacts with NEMO and RIP1 to constitutively activate IKK complex and NF-kappaB: implication in breast cancer metastasis. Oncogene.

[47] Zhao B, Ye X, Yu J, Li L, Li W, Li S (2008). TEAD mediates YAP-dependent gene induction and growth control. Genes Dev.

[48] Zaidi SK, Sullivan AJ, Medina R, Ito Y, van Wijnen AJ, Stein JL (2004). Tyrosine phosphorylation controls Runx2-mediated subnuclear targeting of YAP to repress transcription. Embo J.

[49] Miano JM, Long X, Fujiwara K (2007). Serum response factor: master regulator of the actin cytoskeleton and contractile apparatus. Am J Physiol Cell Physiol.

[50] Wozniak MA, Modzelewska K, Kwong L, Keely PJ (2004). Focal adhesion regulation of cell behavior. Biochim Biophys Acta.

[51] Miller AL, Wang Y, Mooseker MS, Koleske AJ (2004). The Abl-related gene (Arg) requires its F-actin-microtubule cross-linking activity to regulate lamellipodial dynamics during fibroblast adhesion. J Cell Biol.

[52] Kaverina I, Straube A (2011). Regulation of cell migration by dynamic microtubules. Semin Cell Dev Biol.

[53] Ezratty EJ, Partridge MA, Gundersen GG (2005). Microtubule-induced focal adhesion disassembly is mediated by dynamin and focal adhesion kinase. Nat Cell Biol.

[54] Goncharova EA, James ML, Kudryashova TV, Goncharov DA, Krymskaya VP (2014). Tumor suppressors TSC1 and TSC2 differentially modulate actin cytoskeleton and motility of mouse embryonic fibroblasts. PLoS One.

[55] Horton ER, Humphries JD, James J, Jones MC, Askari JA, Humphries MJ (2016). The integrin adhesome network at a glance. J Cell Sci.

[56] Robertson J, Jacquemet G, Byron A, Jones MC, Warwood S, Selley JN (2015). Defining the phospho-adhesome through the phosphoproteomic analysis of integrin signalling. Nat Commun.

[57] Rusan NM, Fagerstrom CJ, Yvon AM, Wadsworth P (2001). Cell cycle-dependent changes in microtubule dynamics in living cells expressing green fluorescent protein-alpha tubulin. Mol Biol Cell.

[58] Etienne-Manneville S (2013). Microtubules in cell migration. Annu Rev Cell Dev Biol.

[59] Rubin CI, Atweh GF (2004). The role of stathmin in the regulation of the cell cycle. J Cell Biochem.

[60] Di Paolo G, Antonsson B, Kassel D, Riederer BM, Grenningloh G (1997). Phosphorylation regulates the microtubule-destabilizing activity of stathmin and its interaction with tubulin. FEBS Lett.

[61] Li N, Jiang P, Du W, Wu Z, Li C, Qiao M (2011). Siva1 suppresses epithelial-mesenchymal transition and metastasis of tumor cells by inhibiting stathmin and stabilizing microtubules. Proc Natl Acad Sci U S A.

[62] Liu F, Sun YL, Xu Y, Wang LS, Zhao XH (2013). Expression and phosphorylation of stathmin correlate with cell migration in esophageal squamous cell carcinoma. Oncol Rep.

[63] Bhardwaj A, Ganesan N, Tachibana K, Rajapakshe K, Albarracin CT, Gunaratne PH (2015). Annexin A1 preferentially predicts poor prognosis of basal-like breast cancer patients by activating mTOR-S6 signaling. PLoS One.

[64] Yang CC, Graves HK, Moya IM, Tao C, Hamaratoglu F, Gladden AB (2015). Differential regulation of the Hippo pathway by adherens junctions and apical-basal cell polarity modules. Proc Natl Acad Sci U S A.

