# Supplementary figures and images for: Phosphoproteomics reveals network rewiring to a pro-adhesion state in annexin-1-deficient mammary epithelial cells
Source: Breast Cancer Res. 2017 Dec 12;19:132. doi: 10.1186/s13058-017-0924-4 (PMC5727667; doi:10.1186/s13058-017-0924-4)

## Additional file 2: Figure S1

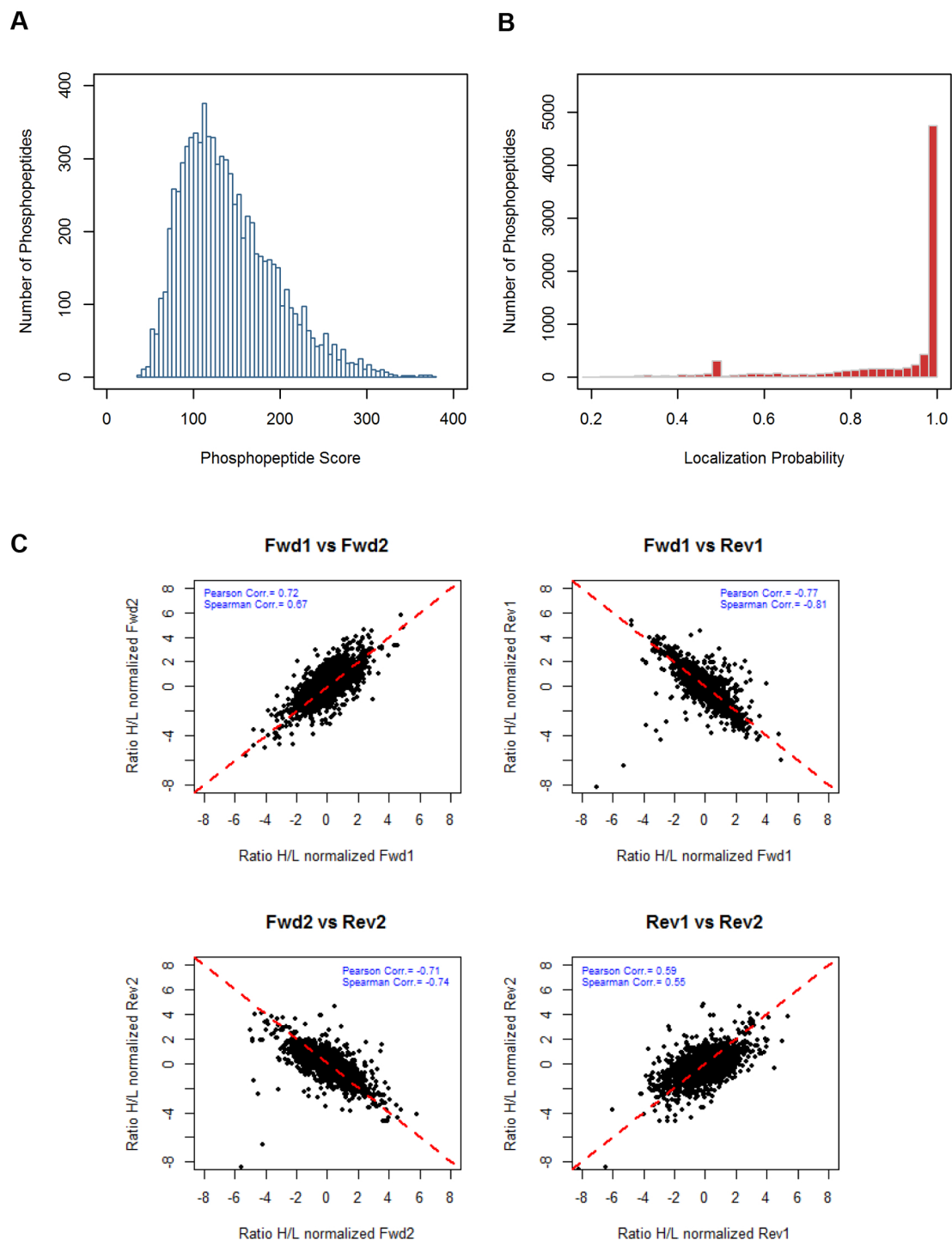

Supplement: Supplementary file 2 — Phosphoproteomic data quality and reproducibility. A Distribution of Andromeda scores of the identified phosphorylated peptides show that most peptides were identified with high scores with a median score of 130. B Distribution of localization probabilities of identified phosphorylation sites. The median probability was ~ 0.97. C Assessment of reproducibility between the four biological replicates carried out with SILAC label swapping shows optimal reproducibility among the forward and reverse experiments. The correlation scores are indicated in each scatter plot. (PDF 1803 kb) [file 13058_2017_924_MOESM2_ESM.pdf]

Additional file 3: Figure S2

A

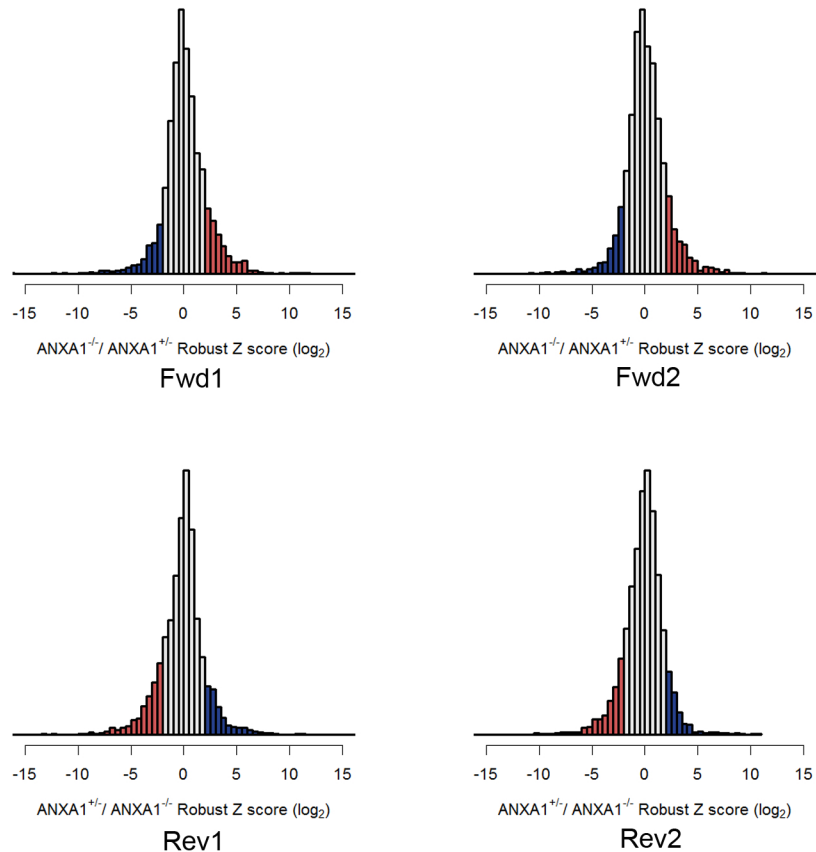

B

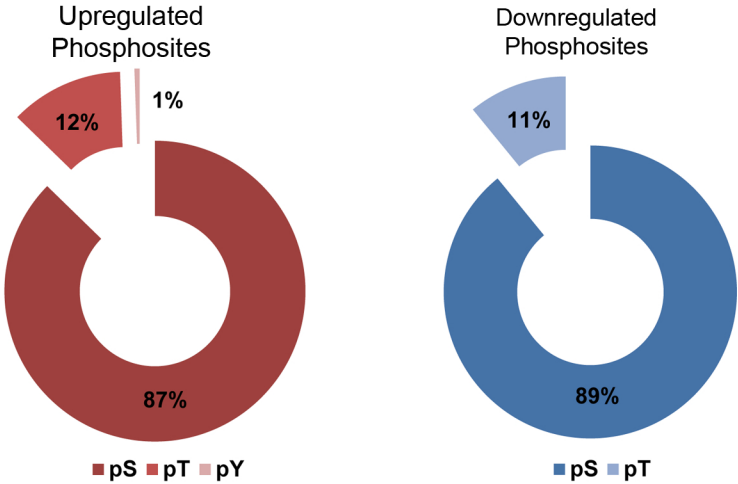

Supplement: Supplementary file 3 — ANXA1-regulated phosphoproteome in mammary epithelial cells. A Distribution of robust z scores of the quantified phosphopeptides. The regions highlighted in red and blue correspond to upregulated and downregulated phosphopeptides, respectively. The sites that were reliably regulated above or below the MAD threshold of ± 2 in three out of four replicates were considered ANXA1-responsive and these at least displayed a 1.8-fold change. The robust z scores were based on log2 normalized fold changes. B Distribution of serine, threonine and tyrosine sites among the regulated phosphorylation sites. (PDF 1059 kb) [file 13058_2017_924_MOESM3_ESM.pdf]

## Additional file 5: Figure S3

**A**

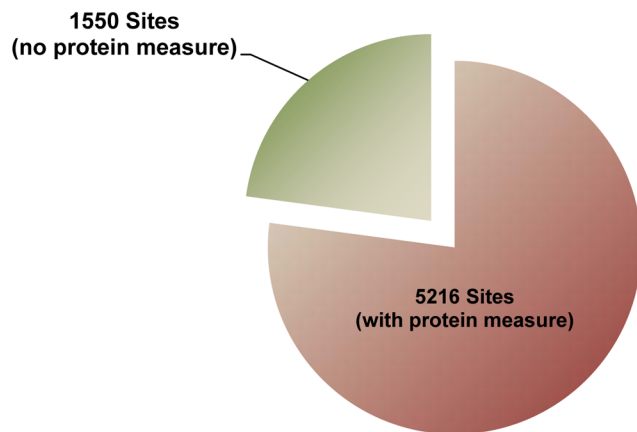

**B**

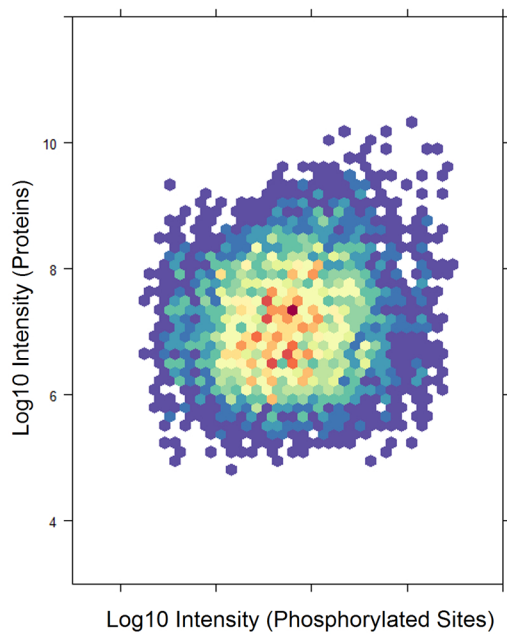

Supplement: Supplementary file 5 — Comparison of quantified proteome and phosphoproteome in ANXA1-deficient mammary epithelial cells. A Number of class I phosphorylation sites with corresponding protein quantification. Except for 1550 sites on 765 proteins that had no corresponding protein measure, the rest of the sites mapped to 1765 proteins with abundance measures. B Intensity-based density plot comparing protein and phosphorylation abundance shows poor correlation. (PDF 1234 kb) [file 13058_2017_924_MOESM5_ESM.pdf]

Additional file 9: Figure S4

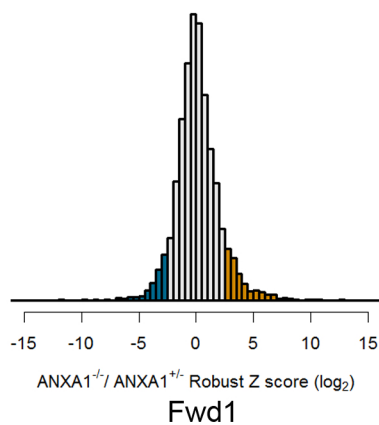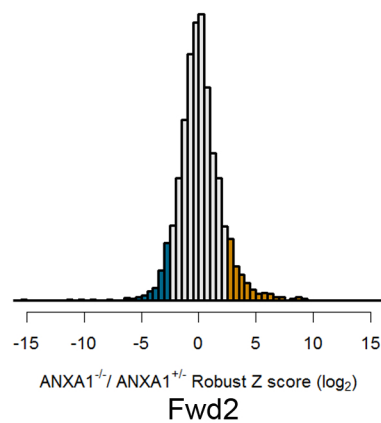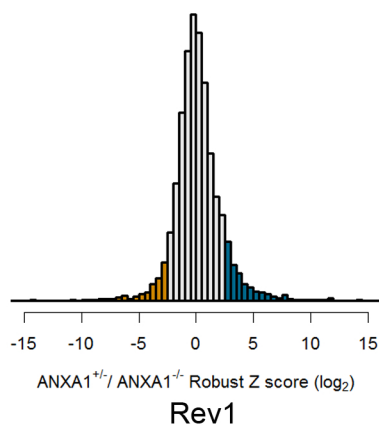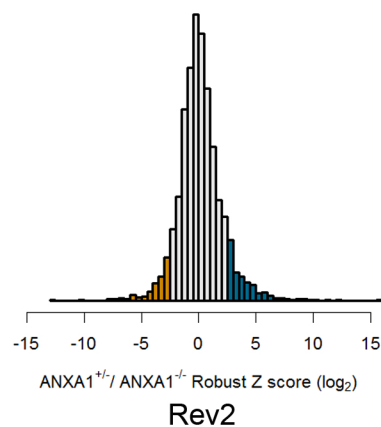

Supplement: Supplementary file 9 — ANXA1-regulated proteins in mammary epithelial cells. Distribution of robust z scores of the quantified proteins. The regions highlighted in orange and blue correspond to upregulated and downregulated phosphopeptides, respectively. Only those proteins regulated in at least three out of four experiments were considered regulated. (PDF 786 kb) [file 13058_2017_924_MOESM9_ESM.pdf]

Additional file 13: Figure S5

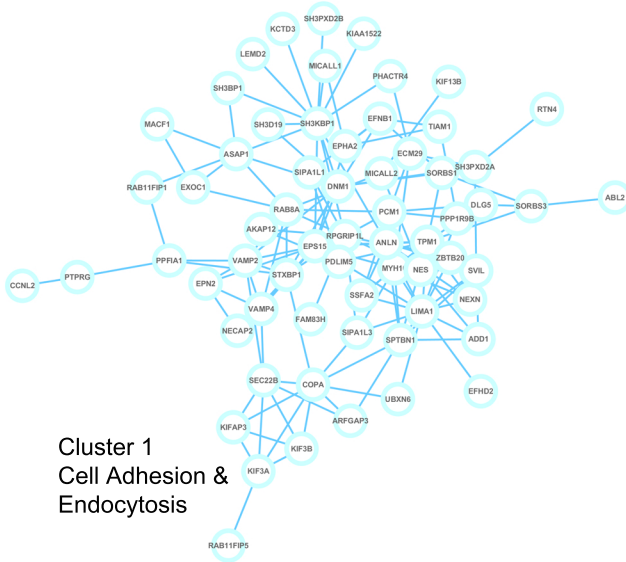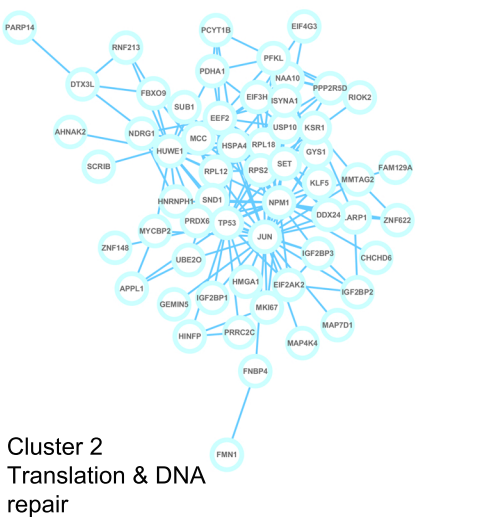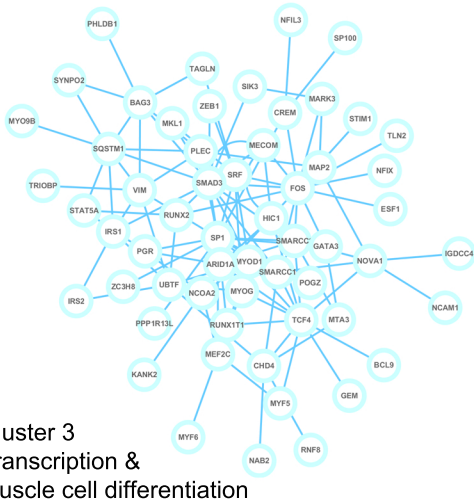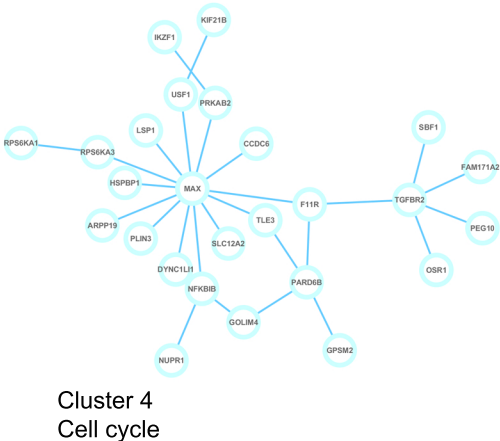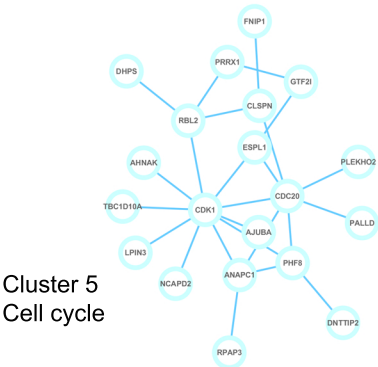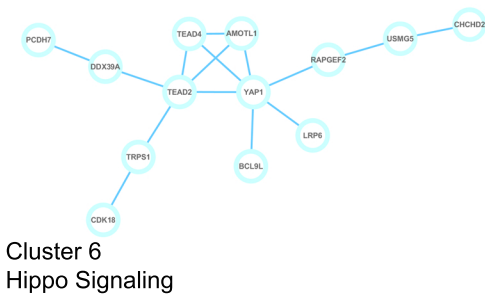

Supplement: Supplementary file 13 — Clusters enriched in ANXA1-regulated protein interaction network. Integrated protein-protein interaction network was constructed using those proteins with ANXA1-responsive phosphorylation changes along with transcription factors predicted from ANXA1-regulated proteome. Clusters were identified using GLay community structure detection and the top clusters identified along with their associated functions are shown. (PDF 5678 kb) [file 13058_2017_924_MOESM13_ESM.pdf]
